# Do Healthcare Professionals Agree with Delphi Expert Recommendations for Instrument Assisted Soft-Tissue Mobilization Precautions and Contraindications? An Exploratory Survey

**DOI:** 10.3390/healthcare13212745

**Published:** 2025-10-29

**Authors:** Scott W. Cheatham, Russell T. Baker

**Affiliations:** 1 Department of Kinesiology, California State University Dominguez Hills, Carson, CA 90747, USA; 2 School of Health and Medical Professions, University of Idaho, Moscow, ID 83844, USA; russellb@uidaho.edu; 3 WWAMI Medical Education Program, University of Idaho, Moscow, ID 83844, USA

**Keywords:** Graston^®^, myofascial, fascia, massage, treatment

## Abstract

Background: Instrument-assisted soft-tissue mobilization (IASTM) is a popular intervention used for myofascial treatment. Healthcare professionals using IASTM must consider precautions and contraindications prior to administering the intervention. A recent international Delphi survey of IASTM experts recommended a list of 39 conditions to be considered as precautions and contraindications. The clinical relevance of these recommendations among healthcare professionals is of interest. The purpose of the survey was to explore healthcare professionals’ agreement regarding the IASTM Delphi recommendations for precautions and contraindications. Methods: A 16-question electronic survey was emailed to members of the Academy of Orthopedic Physical Therapy, American Academy of Sports Physical Therapy, National Athletic Trainers Association, and members of private physical therapy and athletic training Facebook^®^ and LinkedIn™ groups. Survey inclusion criteria included being a healthcare professional who has clinical experience using IASTM with patients. The strength of agreement grade scale was used to explore professionals’ opinions and agreement with the expert recommendations. Results: Four hundred and forty-five professionals (men = 52%; women = 46%; other = 2%) (mean age = 49 ± 12.33 years old) completed the survey. Most respondents (mean = 62%) agreed with 12 of 39 recommended conditions across the strength of agreement grade categories. The conditions included five precautions, four contraindications, and three conditions that could be both. Respondents also listed 32 other conditions they felt were relevant. Discussion: These survey results illustrate diversity among professionals’ agreement with expert recommendations. This may be explained by variations in clinical practice patterns and gaps in the research on this topic. The IASTM Delphi study’s recommended list of precautions and contraindications provides valuable information but is not all-inclusive, as other conditions may exist for different patients. When exploring understudied topics, researchers may want to begin with a Delphi study to establish expert recommendations, followed by an assessment of their clinical relevance through related survey studies of healthcare professionals’ agreement on the topic. Conclusions: This exploratory survey introduced a novel method of assessing the clinical relevance of a Delphi study on IASTM precautions and contraindications among healthcare professionals.

## 1. Introduction

Instrument-assisted soft-tissue mobilization (IASTM) is a popular myofascial intervention used by healthcare professionals to treat different musculoskeletal conditions or to enhance athletic performance [1,2,3,4,5,6]. IASTM has also been used as an intervention to reduce pain, and to improve joint range of motion, soft-tissue mobility, and function [1,6,7,8].

Healthcare professionals using IASTM must consider potential precautions and contraindications prior to administering the intervention [6]. To date, only two peer-reviewed publications have suggested IASTM safety considerations [6,9]. Most of the guidance for IASTM treatment precautions and contraindications has come from manufacturers through their continued education courses [10,11,12,13]. There has been concern regarding the lack of investigation into IASTM safety considerations over the past 17 years by IASTM researchers [14]. This research gap creates a disconnect between research evidence and clinical practice.

To address this research gap, an international panel of IASTM experts participated in a recent modified Delphi study on IASTM precautions and contraindication [14]. Through the three-round Delphi process, the expert panel determined a final list of 39 conditions that can be considered precautions and/or contraindications for IASTM treatment [14]. The rationale for the expert recommendations was to update the existing list of conditions and to encourage further investigations into this topic [14].

Healthcare professionals should consider that Delphi studies are classified as lower-level evidence representing expert opinion and are often used when evidence regarding a specific healthcare topic is limited or unavailable [14,15]. Over the past decade, several Delphi studies on specific myofascial interventions have been published, with notable studies on foam rolling cautions and contraindications [16], the diagnostic criteria of myofascial trigger points [17], and recommendations for stretching [18]. These studies have addressed potential research gaps by providing expert recommendations and insights that will hopefully stimulate more investigations on these topics [14].

Delphi studies have limitations due to potential biases such as the survey questionnaire design, expert panel selection, the consensus criteria for each round, and how surveys are answered [15,19]. These potential biases can challenge the generalizability of the Delphi study recommendations. Despite these limitations, Delphi studies may offer clinically relevant recommendations on an understudied topic, which could be valuable for healthcare professionals.

A potential concern is that the Delphi consensus recommendations have not been validated in real-world clinical practice. To date, there is no published method of measuring the efficacy of expert Delphi recommendations on specific topics among healthcare professionals. This leaves a gap in the understanding of the clinical relevance of such expert recommendations and whether healthcare professionals agree with these expert opinions [16,17,18].

A novel method of evaluating the clinical relevance of myofascial intervention Delphi study recommendations is to investigate the impact of their recommendations among practicing healthcare professionals [20]. This can be accomplished through a survey of healthcare professionals’ opinions regarding Delphi expert recommendations. Healthcare professionals’ agreement with published IASTM Delphi expert recommendations on precautions and contraindications is of interest. The purpose of the survey was to explore healthcare professionals’ agreement regarding the IASTM Delphi recommendations for precautions and contraindications.

## 2. Materials and Methods

### 2.1. Study Design

This cross-sectional descriptive study surveyed healthcare professionals regarding their agreement with recently published IASTM guidelines for 39 conditions classified as precautions and/or contraindications [14]. Healthcare professionals were recruited via convenience sampling between June 2025 and August 2025. Emails were sent to members from the Academy of Orthopedic Physical Therapy (*n* = 13,931), American Academy of Sports Physical Therapy (*n* = 6397), National Athletic Trainers Association (*n* = 5378), and members of private healthcare Facebook^®^ groups and LinkedIn^®^ groups (*n* = 12,350). Prior research has documented that social media platforms are an effective recruitment tool for healthcare research purposes [21,22,23]. It is important to consider that these sampling methods focused on specific groups of healthcare professionals versus a random sample of respondents. These sampling techniques have been used in similar healthcare surveys conducted by the researchers of this study [24,25]. The survey inclusion criteria included being a healthcare professional who has clinical experience using IASTM as an intervention with patients. Respondents were excluded if they did not meet the inclusion criteria. This study was approved by the institutional review board at California State University Dominguez Hills (IRB-FY2025-165).

### 2.2. Survey Development

The online survey (Qualtrics, 333 W. River Park Drive Provo, UT, USA) was developed by the primary researcher (S.W.C) using similar methods as the recently published IASTM Delphi study [14]. This online survey included the list of 39 conditions determined by the panel of IASTM experts in the published study [14]. The goal was to replicate similar survey questions to provide a direct comparison among the published study and these survey results.

The survey consisted of 16 total questions that included one informed consent question and 15 items comprising nominal, multiple-choice, and open text box questions. Initial questions included respondent demographics, IASTM education, and clinical practice patterns. The rest of the survey included three categories of medical conditions: (1) musculoskeletal, cardiorespiratory, and chronic conditions (2) integumentary, connective tissue, nervous system, and psychological conditions (3) miscellaneous conditions and treatment considerations. Survey respondents answered the questions in each category and classified the medical conditions as either precaution, contraindication, or both. After each question, there was an open text box for respondents to provide any comments.

### 2.3. Survey Validation

After the initial survey was completed, the first draft underwent one round of pilot testing with two independent healthcare professionals to establish face validity. The professionals reviewed the survey and provided feedback, and revisions were made. After their feedback, a final set of survey items were identified and approved. Both professionals were licensed physical therapists and athletic trainers with doctorate-level education, multiple IASTM publications, and over 15 years of clinical experience with IASTM. The survey responses obtained through survey development and pilot testing were considered independent and were not included in the main study analysis and results. The final survey was further tested for readability using the Flesch–Kincaid grade level test. The 16 questions in the final survey scored 9.6 on the Flesch–Kincaid grade level test, which indicated the English used in the survey was average reading between the 9th to 10th grade level [26]. This survey development process was used in prior healthcare survey studies conducted by the researchers of this study [24,25]. The final survey reflected the categories and related questions from the published IASTM Delphi study to provide a direct comparison among experts and clinical professionals.

### 2.4. Strength of Agreement Grade Scale

The *strength of agreement grade scale* was adapted from the published IASTM Delphi study for this survey to provide a direct measure of agreement among experts and healthcare professionals on this topic [14]. The scale was used to help rank the strength of consensus among survey respondents for classifying the 39 conditions as precautions, contraindications, or both (Table 1). This scale represents the diversity of agreement that may occur among healthcare professionals on a topic [14]. Some professionals may not agree that specific conditions are absolute precautions or contraindications. Healthcare professionals may have different treatment approaches, philosophies, and beliefs based upon the research evidence [14]. Question items that did not meet or exceed the ≥ 50% agreement threshold were considered to be both a potential precaution and a contraindication. It is recommended that items in grade D (both) are at least considered an IASTM precaution by healthcare professionals [14]. These scale categories were defined in the prior IASTM Delphi study [14] and are supported among other healthcare Delphi studies that used similar ranked agreement categories to measure expert consensus [20,27].

### 2.5. Data Processing

Data were downloaded from the Qualtrics survey platform for analysis. Statistical analysis was performed using Microsoft Excel^®^ (Bellevue, Washington, DC, USA). Descriptive data included total responses, frequency count, and percentages. Data were treated conservatively; any respondents who failed to answer all survey questions were removed from the data set.

## 3. Results

### 3.1. Total Responses and Demographics

**Figure 1:**
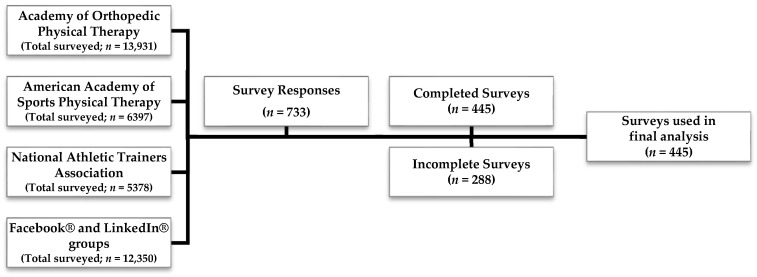
Survey respondents.

Of the 733 participants who responded to the survey, 445 respondents (61%) completed the survey (Figure 1). Two hundred and eighty-eight (39%) incomplete surveys were removed. Only complete surveys were included in the final analysis.

A total of 52% (*n* = 233) of the total respondents were men, while 46% (*n* = 204) were women, and 2% (*n* = 8) fell into other categories. The average age of respondents was 49 years old, ranging from 26 to 83 years. A total of 62% (*n* = 280) of respondents reported being a physical therapist, 28% (*n* = 125) a certified athletic trainer, and 10% reported having other healthcare credentials (Table 2). Respondents reported working in variety of settings, such as an outpatient healthcare facility (47%, *n* = 210); a university or college sports medicine or athletic training facility (10%, *n* = 46); a secondary school athletic training facility (e.g., middle or high school) (9%, *n* = 41); and a hospital-based healthcare facility (7%, *n* = 33). Twenty-seven percent of respondents also reported working in other settings. Respondents reported being a healthcare professional for an average of 21 years (Table 2).

### 3.2. Respondent IASTM Education and Treatment Patterns

For education, most respondents reported collaborating with professional colleagues on IASTM treatment (49%, *n* = 216), reading IASTM peer-review journal articles (43%, *n* = 189), watching online IASTM videos (31%, *n* = 140), and attending online education courses (30%, *n* = 134). Most respondents reported using IASTM during their treatment at least 1× per week (32%, *n* = 141), at least 1× per day (29%, *n* = 130), or at least 1× per month (16%, *n* = 72) (Table 2).

### 3.3. Musculoskeletal, Cardiorespiratory, and Chronic Conditions

For precautions, survey respondents reported *strong* agreement for De Quervain’s tenosynovitis (88%, *n* = 390), Dupuytren’s contracture (81%, *n* = 361), and rheumatoid arthritis (70%, *n* = 313). Respondents also reported *moderate to weak* agreement for lupus (65%, *n* = 289), psoriatic arthritis (58%, *n* = 257), and peripheral vascular disease or insufficiency, and varicose veins (52%, *n* = 231).

For contraindications, survey respondents reported *strong* agreement for unhealed or unstable bone fracture (76%, *n* = 337) and *moderate to weak* agreement for thrombophlebitis or osteomyelitis (69%, *n* = 306), bleeding disorders (e.g., hemophilia) (64%, *n* = 287), and cancer and malignancy (51%, *n* = 225). For *both* precautions and contraindications, respondents chose myositis ossificans (47%, *n* = 209), unhealed bone stress fracture (44%, *n* = 196), and polymyositis (43%, *n* = 192) (Table 3).

### 3.4. Integumentary, Connective Tissue, Nervous System, and Psychological Conditions

For precautions, survey respondents reported *strong* agreement for mild/moderate skin hypersensitivity (86%, *n* = 382) and fibromyalgia with nervous system sensitivity (70%, *n* = 312). Respondents also reported *moderate to weak* agreement for abnormal skin sensation (e.g., numbness, tingling) (65%, *n* = 289), healing surgical scars (60%, *n* = 267), and skin burn scars (58%, *n* = 262).

For contraindications, survey respondents reported *strong* agreement for open skin wounds (81%, *n* = 362) and *moderate to weak* agreement for skin scrapes or blisters (66%, n = 295), acute inflammatory skin conditions (58%, *n* = 260), insect bites of unexplained origin (56%, *n* = 248), severe skin hypersensitivity (55%, *n* = 245), and skin rash (53%, *n* = 236).

For *both* precautions and contraindications (<50% agreement), respondents chose psoriasis (49%, *n* = 218) and a psychological condition that could affect a patient’s response to treatment (48%, *n* = 213) (Table 4).

### 3.5. Miscellaneous Conditions and Treatment Considerations

For precautions, survey respondents reported *strong* agreement with medications such as hormone replacement or fluoroquinolone antibiotics (73%, *n* = 324) and *moderate to weak* agreement for flu or flu-like symptoms (65%, *n* = 289) and medications that alter sensation (57%, *n* = 252).

For contraindications, survey respondents only reported *weak* agreement (50–59%) for acute systemic infection (viral or bacterial), fever, or contagious condition (57%, *n* = 252); direct pressure over face, eyes, body prominences, arteries, veins, or nerves (55%, *n* = 243); allergies to metals, emollients, and latex (52%, *n* = 232); severe pain felt by patient (52%, *n* = 230); insulin pump (e.g., treatment around device) (51%, *n* = 227); and a pacemaker (e.g., treatment around device) (51%, *n* = 226).

For *both* precautions and contraindications (<50% agreement), respondents chose inability to communicate (e.g., language or cognitive issues) (44%, *n* = 197), inability to position body during treatment (48%, *n* = 213), pregnancy (high risk) (48%, *n* = 213), and medications that thin blood (45%, *n* = 200) (Table 5).

### 3.6. Other Conditions Suggested by Respondents

Survey respondents also had a chance to list other conditions (via open text box) they felt should be considered as precautions or contraindications. Respondents listed 32 other conditions, with 14 conditions being novel and the other conditions (*n* = 18) being similar to the conditions listed in the first round of the IASTM Delphi study, which listed 81 potential conditions [14]. The 32 suggested respondent conditions included musculoskeletal conditions (*n* = 6, 19%), cardiovascular, respiratory, and chronic conditions (*n* = 14, 44%), integumentary, connective tissue, nervous system, and psychological conditions (*n* = 9, 28%), and miscellaneous conditions or treatment considerations (*n* = 3, 9%) (Table 6). Respondents’ suggested conditions were not appraised by the *strength of agreement grade scale*.

### 3.7. Summary of Agreement Among IASTM Experts and Survey Respondents

Most survey respondents (mean = 62%; *n* = 276) reported a similar consensus to IASTM Delphi expert recommendations for 12 of the 39 recommended conditions based upon the strength of agreement grade categories. For precautions, respondents reported *strong* agreement with experts for mild/moderate skin hypersensitivity (86%) and rheumatoid arthritis (70%). There was *moderate to weak* agreement among respondents and experts for flu or flu-like symptoms (65%), skin burn scars (58%), and medications that alter sensation (57%) (Table 7).

For contraindications, survey respondents reported *strong* agreement with experts for unhealed or unstable bone fracture (75%), open skin wounds (81%), and *moderate* agreement for bleeding disorders (e.g., hemophilia) (64%). There was *weak* agreement among survey respondents and experts for direct pressure over face, eyes, body prominences, arteries, veins, or nerves (56%). For *both* precautions and contraindications, respondents chose pregnancy (high risk) (48%), inability to communicate (44%), and unhealed bone stress fracture (44%) (Table 8).

## 4. Discussion

This cross-sectional descriptive survey study explored healthcare professionals’ agreement with published Delphi recommendations for IASTM precautions and contraindications. To date, no assessment methods have been reported that explore healthcare professionals’ agreement with Delphi study recommendations for myofascial interventions such as IASTM. This leaves a gap in the understanding of the clinical relevance of these types of studies [15]. This survey was considered exploratory, with the goal of establishing initial data to guide future investigations on this topic.

These survey findings suggest a diversity in opinions and agreement among practicing professionals and IASTM Delphi recommendations, which was demonstrated by the consensus regarding 12 conditions across the *strength of agreement grade scale* categories. The consensus included five precautions, four contraindications, and three conditions that could be both (Table 7 and Table 8). Survey respondents also listed 32 other conditions they felt should be considered as potential IASTM precautions and contraindications. Fourteen conditions were novel, and the rest of the conditions were previously listed in the first round of the IASTM Delphi survey, which included 81 potential conditions [14].

Delphi study recommendations are typically from subject matter experts; these guide clinical practice and may vary from the recommendations of healthcare professionals, who may treat a wider array of patients in traditional clinical settings [6]. Delphi studies are classified as lower-level evidence due to the use of expert opinion, and have potential weaknesses from methodological biases [15,19]. These potential biases can challenge the generalizability of the study, which should be considered when interpreting the results [14].

Professionals should also consider that this survey only required one consensus round of respondent feedback, versus the IASTM Delphi study, which performed three survey consensus rounds to eventually develop the recommended list of 39 conditions [14]. These methodological differences must be considered when interpreting the results for clinical practice.

### 4.1. Practice Implications

Healthcare professionals should consider the diversity in agreement among survey respondents and the IASTM Delphi recommendations. There was a strong consensus for only 12 out of 39 conditions, with professionals suggesting 32 additional conditions. The diversity among healthcare professionals’ agreement may have been related to such factors as their clinical training, patient populations, exposure to listed conditions, and treatment philosophies. These survey findings illustrate the need for further study of IASTM precautions and contraindication, with three relevant points being highlighted.

First, Delphi studies can provide valuable expert insights into a specific healthcare topic, such as IASTM precautions and contraindications [14]. These types of studies often provide clinically relevant recommendations that may be useful in understanding professional practice patterns, designing future research, and ultimately determining best-practice recommendations [15]. Professionals should consider that Delphi survey studies compliment available higher-level evidence on the topic or may be the best evidence available on an understudied topic, serving as a starting point for future research to determine best practices [15]. Currently, the IASTM Delphi study is the best available evidence on this topic [14].

Second, related surveys of healthcare professionals, exploring their agreement with the Delphi recommendations, may help determine their clinical relevance and guide research and best practices. Currently, there is no valid method of exploring the clinical relevance of healthcare Delphi study recommendations [20]. Thus, an initial Delphi study followed by a related survey of professionals’ agreement may provide greater insights into the clinical relevance of a specific healthcare topic. To provide a more direct comparison, both studies should include a similar survey methodology. This survey included one round of consensus, while the Delphi study included three rounds.

Third, Delphi studies and related surveys should include similar outcome measures to provide a relevant comparison. A unique feature of this survey was the exploration of healthcare professionals’ agreement with the IASTM Delphi recommendations using the *strength of agreement grade scale*, which was originally introduced in the Delphi study [14]. The use of this scale in both studies provided insights into the level of agreement among professionals regarding the Delphi study’s recommendations. Other researchers have introduced the inclusion of ordinal-type scales in Delphi studies, which may provide value by statistically calculating and analyzing survey answers [28]. These three points should be considered in future research on this topic.

Overall, clinical professionals should consider the value of the IASTM Delphi expert recommendations for IASTM precautions and contraindication, but should also develop their own comprehensive list of conditions that are relevant to their patient population and treatment philosophy.

### 4.2. Future Research

These survey results suggest a need for future higher-level controlled studies on IASTM precautions and contraindications, since this topic is understudied. The diversity among healthcare professionals and Delphi recommendations was evident and warrants further study. To date, there are no higher-level controlled studies that have directly investigated IASTM treatment safety [6,9,14]. Ideally, higher-level controlled IASTM studies should include randomized controlled trials using different treatment parameters among subjects with known medical conditions and a comparison group, using outcomes such as joint range of motion, pain modulation, and movement efficiency [6]. This will help to determine the best IASTM safety practices.

Future investigations on understudied healthcare topics should begin with a Delphi study, followed by a related survey of professionals to explore agreement and clinical relevance. Both studies should have a similar survey methodology (e.g., similar survey rounds) and outcomes such as the *strength of agreement grade scale* or other validated Delphi scales [14,28]. Researchers should strive to find valid methods for measuring the relevance of Delphi recommendation. This survey was an initial attempt to explore the use of the proposed methodology for future higher-level investigations.

### 4.3. Limitations

Several limitations of this study need to be discussed. First, this survey was sent to a group of identified healthcare professionals from different organizations and social media groups, which can be considered a form of selection bias. A larger, more diverse sample of professionals who use IASTM in clinical practice may have produced different results. However, to the researchers’ knowledge, this is the first survey study using this methodology to explore agreement among professionals and the published expert recommendations on IASTM precautions and contraindications. Future studies should sample larger, diverse populations among different countries to provide a broader perspective on this topic. Second, the study results can only be generalized to the healthcare professionals surveyed. Other healthcare professionals or those who work with specialized patient populations may have provided different responses that are more applicable to those patients or professional settings. Third, the survey contained 39 recommended medical conditions from a published Delphi study. A more robust list of all considered medical conditions, as opposed to only those that reached consensus among experts, may have revealed different insights on what healthcare professionals consider potential IASTM precautions and/or contraindications in comparison to expert consensus. This limitation was further revealed when respondents included other conditions within the open text box that they felt were relevant. These responses were not directly compared to the original list from the IASTM Delphi study. Future research should explore the clinical relevance of a more robust list of IASTM’s potential precautions and contraindication among both experts and healthcare professionals. Fourth, our survey documented responses from a combined group of healthcare professionals, without any subgroup analysis by profession, treatment philosophy, years of experience with IASTM, or frequency of IASTM use. Future survey studies with similar subgroup analyses may reveal further insights into specific healthcare professional survey choices. Fifth, this survey required only one round of consensus, while the IASTM Delphi study required three rounds, which may have influenced the final list of conditions. Future investigations should include a similar methodology for both studies to create a more relevant comparison of outcomes.

## 5. Conclusions

This survey was exploratory and provided initial data on how surveys of healthcare professionals can be used to explore the clinical relevance of Delphi study recommendations on a specific topic or to gain further insight into how an intervention is viewed across the wider professional body that implements an intervention in clinical practice. To date, there has been no proposed method of evaluating the clinical relevance of Delphi expert recommendations. Due to their growing popularity in healthcare, there is a need to develop Delphi study assessment methods to determine their clinical relevance among professionals and to assess how differences between expert recommendations and those found in common clinical practice influence patient outcomes.

These survey results revealed the diversity in healthcare professionals’ agreement with the IASTM Delphi expert recommendations for precautions and/or contraindications, which is an understudied topic. Healthcare professionals should consider that the IASTM Delphi recommendations are not all-inclusive but can be considered essential precautions and contraindications for professional practice [14]. The results of this survey suggest that professionals may not always agree with experts on specific IASTM precautions and contraindications. Healthcare professionals agreed with only 12 out of 39 conditions. However, these survey results should encourage professionals to consider expert recommendations while also developing their own comprehensive list of precautions and contraindications for their patients.

Future studies are needed to further develop and update IASTM safety standards. When exploring understudied topics, researchers may want to initially begin with a Delphi study to establish expert recommendations before assessing the clinical relevance of the recommendations through related survey studies of healthcare professionals using similar methodology.

## Figures and Tables

**Table 1:** Strength of agreement grade scale.

Grade	Definition
A (Strong)	>70% agreement
B (Moderate)	69–60% agreement
C (Weak)	59–50% agreement
D (* Both)	<50% agreement

* Both: Condition can potentially be a precaution or contraindication.

**Table 2:** Healthcare professional demographics.

Respondents (N = 445)	Frequency % (N)
**Please describe your “self-identified “gender.**	
Men	52.36% (233)
Women	45.84% (204)
Prefer not to say	01.57% (07)
Non-binary/third gender	00.23% (01)
**Please state your chronological age.**	
Age range (average)	49.33 ± 12.33 years
**Please state how long you have been a healthcare professional.**	
Years (average)	21.39 ± 12.95 years
**Please choose your primary occupation(s) (e.g., credentials) within the healthcare and fitness industry. Choose all that apply.**	
Physical therapist	61.54% (280)
Licensed/certified athletic trainer	28.01% (125)
Chiropractor	04.27% (19)
Physical therapist assistant	02.47% (11)
Massage therapist	00.67% (3)
Nurse practitioner or nurse	00.45% (2)
Occupational therapist	00.22% (1)
Physician assistant	00.22% (1)
Acupuncturist	00.22% (1)
Other	1.00% or less (3)
**Please choose your primary work setting(s). Choose all that apply.**	
Outpatient healthcare facility (e.g., physical therapy, chiropractic)	47.19% (210)
University or college sports medicine or athletic training facility	10.34% (46)
Secondary school athletic training facility (e.g., middle or high school)	09.21% (41)
Hospital or hospital-based healthcare facility	07.42% (33)
Academic or research faculty	06.74% (30)
Academic faculty clinic	02.92% (13)
Industrial/occupational health services	02.92% (13)
Professional sports/Sports performance facility	02.47% (11)
In-home services	02.25% (10)
Physician owned practice	01.80% (8)
Military service facility	01.35% (6)
Other	05.39% (24)
**What type of IASTM education have you participated in within the past 10 years? Choose all that apply.**	
Collaborated with professional colleagues on IASTM treatment	48.54% (216)
Read IASTM peer-reviewed journal articles	42.84% (189)
Attended traditional (in-person) education courses	41.80% (186)
Watched online IASTM videos (e.g., YouTube, social media)	31.46% (140)
Attended online education courses	30.11% (134)
No participation in IASTM education	15.06% (67)
Other	03.15% (14)
**How frequently do you use IASTM during your patient treatments? Choose one answer.**	
At least 1× per week	31.70% (141)
At least 1× per day	29.21% (130)
At least 1× per month	16.18% (72)
Never use	10.56% (47)
At least 1× in a half of a year	10.34% (46)
Other	02.01% (9)

**Table 3:** Musculoskeletal, cardiorespiratory, and chronic conditions.

	Precautions		Contraindications	
(N = 445)	Conditions	Agreement (%)	Conditions	Agreement (%)
**A (Strong)** (≥70% agreement)	- De Quervain’ s tenosynovitis	87.64% (390)	- Unhealed or unstable bone fracture	75.73% (337)
	- Dupuytren’s contracture	81.12% (361)		
	- Rheumatoid arthritis	70.33% (313)		
**B (Moderate)** (69–60% agreement)	- Lupus	64.94% (289)	- Thrombophlebitis or osteomyelitis	68.76% (306)
			- Bleeding disorders (e.g., hemophilia)	64.49% (287)
**C (Weak)** (59–50% agreement)	- Psoriatic arthritis	57.80% (257)	- Cancer and malignancy	50.56% (225)
	- Peripheral vascular disease or insufficiency, varicose veins	51.91% (231)		
* D (Both) (<50% agreement)	**Conditions**		**Agreement (%)**	
	- Myositis ossificans		47.00% (209)	
	- Unhealed bone stress fracture		44.04% (196)	
	- Polymyositis		43.15% (192)	

* D (Both): Condition can potentially be a precaution and contraindication.

**Table 4:** Integumentary, connective tissue, nervous system, and psychological conditions.

	Precautions		Contraindications	
(N = 445)	Conditions	Agreement (%)	Conditions	Agreement (%)
**A (Strong)** (≥70% agreement)	- Mild/moderate skin hypersensitivity	85.84% (382)	- Open skin wounds	81.35% (362)
	- Fibromyalgia with nervous system sensitivity	70.11% (312)		
**B (Moderate)** (69–60% agreement)	- Abnormal skin sensation (e.g., numbness, tingling)	64.94% (289)	- Skin scrapes or blisters	66.30% (295)
	- Healing surgical scars	60.00% (267)		
**C (Weak)** (59–50% agreement)	- Skin burn scars	57.88% (262)	- Acute inflammatory skin conditions	58.43% (260)
			- Insect bites of unexplained origin	55.73% (248)
			- Severe skin hypersensitivity	55.06% (245)
			- Skin rash	53.03% (236)
* D(Both) (<50% agreement)	**Conditions**		**Agreement (%)**	
	- Psoriasis		48.99% (218)	
	- Psychological condition that would affect a patient’s response to treatment		47.86% (213)	

* D (Both): Condition can potentially be a precaution and contraindication.

**Table 5:** Miscellaneous conditions and treatment considerations.

	Precautions		Contraindications	
(N = 445)	Conditions	Agreement (%)	Conditions	Agreement (%)
**A (Strong)** (≥70% agreement)	- Medications such as hormone replacement or fluoroquinolone antibiotics	72.80% (324)		
**B (Moderate)** (69–60% agreement)	- Flu or flu-like symptoms	64.94% (289)		
**C (Weak)** (59–50% agreement)			- Acute systemic infection (viral or bacterial), fever, or contagious condition	56.62% (252)
	-Medications that alter sensation	56.62% (252)	- Direct pressure over face, eyes, body prominences, arteries, veins, or nerves	54.61% (243)
			- Allergies to metals, emollients, and latex	52.13% (232)
			- Severe pain felt by patient	51.68% (230)
			- Insulin pump (e.g., treatment around device)	51.01% (227)
			- Pacemaker (e.g., treatment around device)	50.78% (226)
* D (Both) (<50% agreement)	**Conditions**		**Agreement (%)**	
	- Inability to communicate (e.g., language or cognitive issues)		44.23% (197)	
	- Inability to position body during treatment		47.87% (213)	
	- Pregnancy (high risk)		47.86% (213)	
	- Medications that thin blood		44.94% (200)	

* D (Both) category = respondents did not clearly classify the condition as a precaution or contraindication.

**Table 6:** Other conditions suggested by respondents.

Respondent Suggestions for Other Conditions	Condition Listed in IASTM Delphi Study
- Abnormal skin sensation (e.g., numbness)	Yes
- Acute inflammatory skin conditions	Yes
- Acute and chronic ligament sprain	Yes
- Acute and chronic muscle strain, and/or tendon injury	Yes
- Acute contusion	**No**
- Ankylosing spondylitis	Yes
- Anemia	**No**
- Cancer or malignancy	Yes
- Connective tissue disorders (e.g., Ehlers–Danlos Syndrome, Hypermobility Spectrum Disorders)	Yes
- Heart disease	Yes
- Healing surgical scars	Yes
- Hemophilia	**No**
- Hypertension (uncontrolled)	Yes
- Infection (e.g., meningitis, encephalitis)	Yes
- Local joint or tissue inflammation	Yes
- Muscular dystrophy	**No**
- Diabetes mellitus	**No**
- Lymphedema	Yes
- Lipoma	No
- Medications that thin blood	Yes
- Neuropathy	**No**
- Osteoporosis	Yes
- Peripheral vascular disease/insufficiency, varicose veins	Yes
- Post radiation fibrosis	**No**
- Raynaud’s disease	**No**
- Recent surgery	**No**
- Rhabdomyolysis	**No**
- Skin fungal or bacterial infection (e.g., cellulitis)	**No**
- Stroke	Yes
- Tendon rupture/unhealed tendon repair	No
- Thrombophlebitis or osteomyelitis	Yes
- Scleroderma	**No**

**Table 7:** Precautions: summary of agreement.

Strength of Agreement	IASTM Experts	Healthcare Professionals	Consensus Agreement
**A (Strong)** (≥70% agreement)	- Mild/moderate skin hypersensitivity	- Mild/moderate skin hypersensitivity	**Yes**
	- Rheumatoid arthritis	- Rheumatoid arthritis	**Yes**
**B (Moderate)** (69–60% agreement)	- Flu or flu-like symptoms	- Flu or flu-like symptoms	**Yes**
	- Skin burn scars	- Skin burn scars	**Yes**
**C (Weak)** (59–50% agreement)	- Medications that alter sensation	- Medications that alter sensation	**Yes**

**Table 8:** Contraindications and both precautions/contraindications: summary of agreement.

Strength of Agreement	IASTM Experts	Healthcare Professionals	Consensus Agreement
**A (Strong)** (≥70% agreement)	- Unhealed or unstable bone fracture	- Unhealed or unstable bone fracture	**Yes**
	- Open skin wounds	- Open skin wounds	**Yes**
**B (Moderate)** (69–60% agreement)	- Bleeding disorders	- Bleeding disorders	**Yes**
**C (Weak)** (59–50% agreement)	- Direct pressure over face, eyes, body prominences, arteries, vein, or nerves	- Direct pressure over face, eyes, body prominences, arteries, veins, or nerves	**Yes**
**Precautions and Contraindications**			
* D (Both) (<50% agreement)	- Pregnancy (high risk)	- Pregnancy (high risk)	**Yes**
	- Inability to communicate	- Inability to communicate	**Yes**
	- Unhealed bone stress fracture	- Unhealed bone stress fracture	**Yes**

* D (Both) category = respondents did not clearly classify the condition as a precaution or contraindication.

## Data Availability

The raw data supporting the conclusions of this article will be made available by the authors on request.
